# Enantiomeric Ratio Modulates Hierarchical Networks and Rheological Performance in Cyclohexane Bisurea Supramolecular Gels

**DOI:** 10.3390/gels11100821

**Published:** 2025-10-13

**Authors:** Shaoshuai Hua, Yuqian Jiang, Andong Song, Jian Jiang

**Affiliations:** CAS Key Laboratory of Nanosystem and Hierarchical Fabrication, National Center for Nanoscience and Technology, Beijing 100190, China; huashaoshuai1995@163.com (S.H.); songad2021@nanoctr.cn (A.S.)

**Keywords:** supramolecular gel, enantiomeric ratio modulates, cyclohexane bisurea

## Abstract

This study presents an enantiomeric-ratio-driven strategy for constructing mechanically robust supramolecular gels using cyclohexane bisurea derivatives. By employing non-equimolar enantiomeric mixtures, we achieved an ultralow critical gelation concentration (CGC < 2 mg/mL) in toluene, representing a reduction of more than fivefold compared to homochiral single-enantiomer systems. Rheological measurements revealed substantially enhanced mechanical properties in the non-equimolar gels, with yield stress and storage modulus values up to 17 and 20 times higher, respectively, than those of single-enantiomer gels. Morphological analyses (SEM and POM) indicated that pure enantiomers form isolated crystalline fibers with limited connectivity, whereas racemic mixtures yield disordered amorphous aggregates. In contrast, non-equimolar mixtures self-assemble into hierarchical “sea urchin-like” architectures, wherein crystalline fibers radiate from central cores to form densely interconnected networks. This unique structural motif underpins both the ultralow CGC and superior mechanical performance. Complementary FT-IR, XRD, and DSC analyses demonstrated that chiral imbalance modulates hydrogen-bonding interactions and structural order, while molecular dynamics (MD) simulations provided insight into the divergent self-assembly pathways among homochiral, racemic, and non-equimolar systems. This work provides a stereochemically guided approach for designing high-performance supramolecular gels with tailored hierarchical structures and enhanced functionality.

## 1. Introduction

Supramolecular gels, formed via the self-assembly of low-molecular-weight gelators (LMWGs), constitute a versatile category of soft materials characterized by stimuli-responsive behavior and tunable properties [1,2,3,4,5,6].These distinctive characteristics render them promising candidates for applications in diverse fields such as drug delivery [7,8,9,10,11,12], sensing [13,14,15,16,17,18,19], and flexible electronics [20,21,22,23,24,25]. Unlike polymer gels, supramolecular gels form three-dimensional (3D) networks that immobilize solvents through reversible non-covalent interactions, including hydrogen bonding, van der Waals forces and π–π stacking [26,27,28,29,30]. The formation of these networks typically begins with nucleation, proceeds through elongation into primary assemblies, and ultimately culminates in the development of spatially organized 3D architectures [31,32,33,34,35,36,37]. These structural frameworks directly govern key rheological properties, such as storage modulus, thixotropy, and stimuli responsiveness, thereby underscoring the critical role of network topology in determining gel functionality [38,39,40,41,42,43,44].

Given the structure-property relationship in gels, understanding and controlling the architecture of self-assembled networks is essential for tailoring their performance. To this end, researchers have utilized various external stimuli (e.g., temperature and pH) in conjunction with diverse gelation methods to modulate the process of network formation [45,46]. For instance, Wang et al. demonstrated a temperature-driven transition between spherulitic and fibrillar networks in molecular gels, enabling precise rheological control [47]. Adams et al. utilized different triggers (e.g., salt, acid, or solvent) with the gelator 2NapFF to fabricate hydrogels with microstructures ranging from aligned fibers and entangled nanofibers to spherulitic domains, each of which exhibits distinct mechanical properties [48]. In another study, George et al. employed surface-catalyzed secondary nucleation to promote fiber branching and bundling in calcium cholate-based hydrogels, leading to a significant enhancement in storage modulus through denser physical crosslinking [49]. Despite these significant advances, achieving efficient and precise control over the hierarchical architecture of supramolecular networks remains a fundamental challenge in this field.

Chiral cyclohexane-based bisurea and bisamide derivatives, pioneered by Hanabusa, Feringa, and Esch, represent a prominent class of LMWGs; these gelators rely on hydrogen bonding and hydrophobic interactions to gel a broad range of polar and apolar solvents [50,51,52,53,54]. However, most existing studies have focused on homochiral cyclohexane derivatives or racemic mixtures, leaving the influence of non-equimolar enantiomeric mixtures on gelation propensity and the resulting network topology largely unexplored.

**Figure 1:**
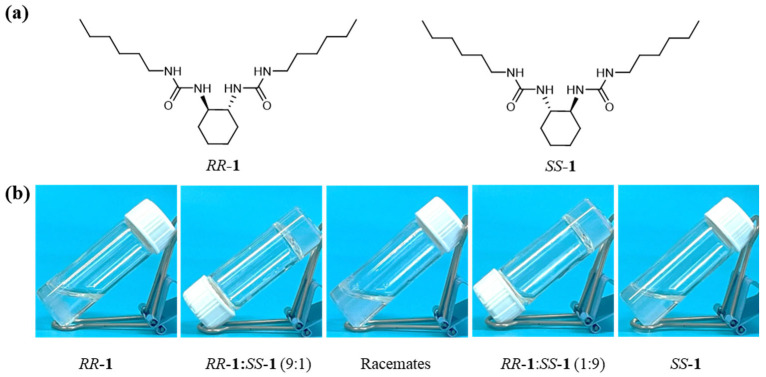
(**a**) Molecular structures of *RR*-**1** and *SS*-**1**. (**b**) Photographs of the samples, presented from left to right, were obtained at a concentration of 2 mg/mL in toluene, with *RR*-**1**:*SS*-**1** ratios of 10:0, 9:1, 5:5, 1:9, and 0:10. Only the 9:1 (1:9) systems can form stable gels.

In response to this research gap, the present work reports an enantiomeric-ratio-driven strategy for fabricating mechanically robust supramolecular gels. Using non-equimolar mixtures of *RR*-**1** and *SS*-**1** enantiomers, we achieved an ultralow critical gelation concentration (CGC < 2 mg/mL) in toluene, representing a more than fivefold reduction compared to the CGC values of homochiral *RR*-**1** or *SS*-**1** systems (Figure 1). Rheological measurements revealed that gels formed from these non-equimolar mixtures exhibit significantly enhanced mechanical strength: the yield stress and storage modulus are up to seventeen and twenty times higher than those of gels based on a single enantiomer, respectively. *RR*-Morphology characterization further confirmed that gels formed from pure enantiomers consist of isolated crystalline fiber-packed networks, whereas racemate-based gels are composed of disordered amorphous aggregates, both systems exhibit poor interconnectivity. In contrast, non-equimolar *RR*-**1**/*SS*-**1** mixtures self-assemble into hierarchical “sea urchin-like” structures, which are characterized by crystalline fibrous assemblies radiating from a central core. These structures subsequently develop into densely packed networks with superior mechanical integrity, a feature that not only accounts for the enhanced mechanical properties of non-equimolar *RR*-**1**/SS-**1** mixtures but also explains the ultralow CGC. Thus, this work establishes a stereochemistry-guided approach for constructing high-strength supramolecular gels at low concentrations, providing a valuable strategy for the rational design of advanced soft materials.

## 2. Results and Discussion

### 2.1. Optimal Condition for Cyclohexane Bisurea Gels

The gelation behavior of *RR*-**1** and *SS*-**1** in toluene exhibited a pronounced and systematic dependence on both concentration and enantiomeric composition. The pure enantiomers formed stable gels only at concentrations exceeding 10 mg/mL, which is consistent with previously reported results (Figure 1, Table S1) [54]. In contrast, non-equimolar mixtures of *RR*-**1** and *SS*-**1** achieved efficient gelation at significantly lower concentrations, indicating a clear correlation between enantiomeric imbalance and enhanced gelation efficiency. Specifically, mixtures with *RR*-**1**:*SS*-**1** ratios of 9:1 and 1:9 displayed the lowest critical gelation concentration (CGC) of approximately 1.5 mg/mL (Table S1). Similarly, systems with ratios of 8:2 and 2:8 also yielded comparably low CGC values of 1.5 mg/mL (Table S1). As the enantiomeric ratio approached equimolar proportions, a progressive increase in CGC was observed: mixtures with ratios of 7:3 and 3:7 exhibited CGC of about 2.5 mg/mL, while those with ratios of 6:4 and 4:6 reached CGCs of 3 mg/mL. The racemic system, in turn, showed a CGC of 4 mg/mL, still lower than that of the homochiral systems (Table S1). These results underscore chiral imbalance as a key structural factor enabling low-concentration gelation in this system, suggesting a distinct supramolecular assembly pathway favored by heterochiral interactions between *RR*-**1/***SS*-**1**.

### 2.2. Gel Morphology Characterizations

**Figure 2:**
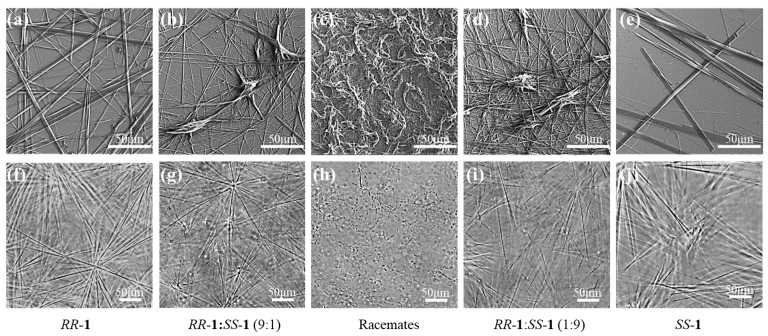
SEM images of xerogels (10 mg/mL) derived from: (**a**) the enantiopure *RR*-**1**; (**b**) the non-equimolar mixture (*RR*-**1**:*SS*-**1** = 9:1); (**c**) the racemic mixture; (**d**) the non-equimolar mixture (*RR*-**1**:*SS*-**1** = 1:9); (**e**) the enantiopure *SS-***1**. For in situ POM images (all samples at 2 mg/mL), obtained from: (**f**) the enantiopure *RR*-**1**; (**g**) the non-equimolar mixture (*RR*-**1**:*SS*-**1** = 9:1); (**h**) the racemic mixture; (**i**) the non-equimolar mixture (*RR*-**1**:*SS*-**1** = 1:9); (**j**) the enantiopure *SS-***1**.

The gel morphology was investigated using scanning electron microscopy (SEM) and polarizing optical microscopy (POM). SEM images of xerogels derived from *RR*-**1** or *SS*-**1** showed that the pure enantiomers self-assembled into highly ordered crystalline fibers, typically hundreds of micrometers in length and with diameters ranging from hundreds of nanometers to several micrometers (Figure 2a,e). Notably, these homochiral fibers exhibited no observable crosslinking junctions between adjacent fibers (Figure 2a,e). In stark contrast, non-equimolar *RR*-**1**/*SS*-**1** mixtures self-assembled into hierarchically organized fibrous networks featuring well-defined junction points, from which dense fibrous bundles radiated outward from distinct nucleation cores (Figure 2b,d and Figure S1). The architecture of these networks displayed a systematic dependence on the enantiomeric ratio: junction density increased progressively as the *RR*-**1**:*SS*-**1** ratio shifted from 9:1 or 1:9 toward 5:5 (Figure S1). At near-racemic compositions (6:4 and 4:6), the junctions transitioned into nodes dominated by amorphous aggregates, while partially retaining fibrous connectivity. In contrast, racemic systems (5:5) primarily formed irregular amorphous aggregates accompanied by only sporadic and poorly defined fibrillar structures (Figure 2c).

Complementary POM analysis further elucidated the divergent assembly processes underlying these structural differences. In pure enantiomer solutions (*RR*-**1** or *SS*-**1**) at sub-gelation concentrations (2 mg/mL), cooling induced instantaneous and homogeneous crystallization, producing large, stacked crystalline fibers within seconds (Figure 2f,j and Video S1). However, these structures developed only ephemeral and weak junctions, resulting in poor network continuity that ultimately led to gravitational settling and precipitation. In contrast, systems with non-equimolar ratios followed a distinct nucleation-elongation pathway: upon cooling, discrete nucleus-like aggregates formed initially and served as structural templates. From these cores, crystalline fibers grew radially and divergently, progressively evolving into hierarchical “sea urchin-like” superstructures (Figure 2g,i and Video S2, *RR*-**1**:*SS*-**1** = 9:1/1:9). Over time, these radiating fibers thickened, interconnected, and ultimately percolated throughout the entire volume. Notably, junction density increased while fiber density decreased as the composition approached racemic parity (Figure S2 and Table S2). In the racemic mixture, exclusively amorphous aggregates formed without evident fibers being observed (Figure 2h and Video S3).

These combined microscopy observations collectively support a primary mechanistic framework for the enantiomeric ratio-dependent gelation behavior. Pure enantiomers (*RR*-**1** or *SS*-**1**) self-assembled into isolated crystalline fibers that lack persistent junctions, which is unfavorable for three-dimensional network formation and accounts for the high critical gelation concentration (CGC ≥ 10 mg/mL) required for macroscopic gelation. In contrast, the racemic system formed interconnected amorphous aggregates rather than long-range ordered fibers, resulting in a less stiffness gel network despite having a lower CGC (4 mg/mL). In non-equimolar systems, chiral imbalance enabled synergistic assembly through stabilized heterochiral junctions: the resulting “sea urchin-like” architecture produced interconnected radiating networks with high junction density and mechanical rigidity, thereby maximizing solvent confinement efficiency and facilitating gelation at ultralow concentrations. The optimal gelation efficiency observed at high enantiomeric excess (*RR*-**1**:*SS*-**1** = 9:1/1:9) correlates with high fiber density radiating from limited nucleation sites, achieving an optimal balance between nucleation and growth for the formation of a robust network.

### 2.3. Rheological Properties Characterizations

Since network topology is a pivotal determinant of gelation and mechanical performance in supramolecular systems, we investigated the rheological properties of gels formed from the pure enantiomer, the racemic mixture, and non-equimolar mixture (*RR*-**1**:*SS*-**1** = 9:1 as a representative case). All samples were prepared at a concentration of 20 mg/mL in toluene to ensure stable gel formation across all compositions, thereby enabling direct structure-rheology correlations.

**Figure 3:**
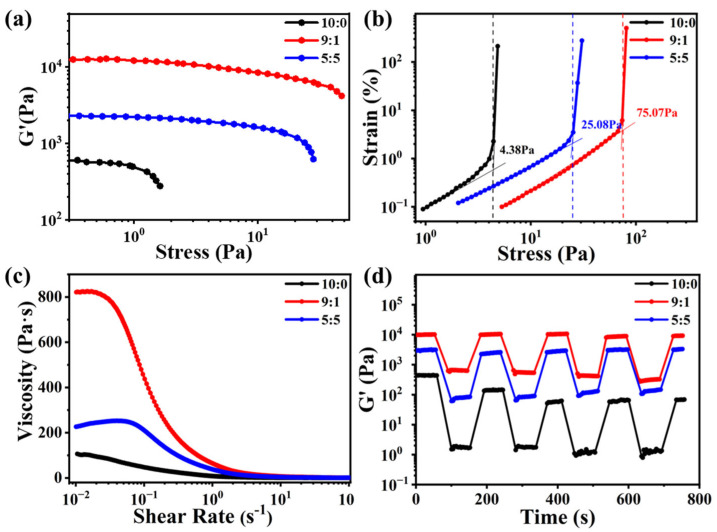
(**a**) Plots of storage modulus G′ versus stress; (**b**) plots of yield stress under strain; (**c**) plots of viscosity versus shear rate; (**d**) shear recovery tests for three gel systems with *RR*-**1**:*SS*-**1** ratio as 10:0, 9:1 and 5:5, respectively.

The storage modulus (G′) exhibited pronounced composition-dependent differences (Figure 3a). The pure enantiomeric gel (10:0) exhibited a G′ of approximately 595.6 Pa, while the racemic gel (5:5) reached around 2280 Pa. Remarkably, the non-equimolar gel (*RR*-**1**:*SS*-**1** = 9:1) achieved a G′ of 12,820 Pa, over 20 times higher than that of the pure enantiomeric system and 5 times greater than that of the racemic gel, three independent rheological experiments confirmed the results are reliability (Figure S4). The G′ decreased as the composition approached racemic parity (Figure S5). This significant enhancement correlates directly with the “sea urchin-like” hierarchical architecture observed via microscopy, which forms a highly interconnected and rigid scaffold. In contrast, the isolated crystalline fibers formed by pure enantiomers (with poor inter-fiber connectivity) and disordered amorphous aggregates formed by racemic mixtures (with less stiffness network) both limit elastic reinforcement. It should be noted that the same trend is also observed for samples at a concentration of 5 mg/mL (Figure S6), which means the remarkable enhancement of the non-equimolar gel *RR*-**1**:*SS*-**1** = 9:1 is robust across various concentrations.

Yield stress exhibited a trend dependent on the enantiomeric ratio that aligns with the storage modulus results: pure enantiomeric gels yielded at approximately 4.38 Pa, racemic gels at about 25.08 Pa, and the non-equimolar gel (*RR*-**1**:*SS*-**1** = 9:1) at 75.07 Pa, representing an approximately 17-fold increase compared to the pure enantiomeric system (Figure 3b). This pronounced enhancement can be attributed to the dense junction points and radially oriented fibers within the sea urchin-like network, which collectively form a robust energy-dissipating framework resistant to deformation. In contrast, the pure enantiomeric gel system, composed of isolated fibers, fails to establish a cohesive network capable of efficient stress distribution, while the racemic gel, consisting of amorphous aggregates, lacks structural anisotropy and reinforcing pathways, resulting in inferior yield resistance.

All gels exhibited non-Newtonian shear-thinning behavior (Figure 3c), with viscosity decreasing sharply as the shear rate increased. Similar to the trends observed for storage modulus and yield stress, the enantiomeric ratio also governed the magnitude of viscosity: at a shear rate of 0.1 s^−1^, pure enantiomeric gels displayed the lowest viscosity (46.6 Pa·s), followed by racemic gels (209.3 Pa·s), and finally the non-equimolar gel (437.1 Pa·s). At 1 s^−1^, the viscosity values decreased to 8.1 Pa·s, 37.9 Pa·s, and 64.6 Pa·s, respectively. The markedly higher viscosity of the non-equimolar gel originates from its sea urchin-like network architecture, which imposes substantial resistance to flow.

Thixotropic behavior, assessed through cyclic shear deformation, revealed pronounced differences in network reversibility among the gel systems (Figure 3d). After the first high-shear cycle, the storage modulus (G′) of pure enantiomeric gels recovered only 32.10% of its initial value, declining to approximately 14% after four cycles. This poor recovery suggests that the stacked fiber network is readily disrupted under shear and that the isolated fibers are unable to efficiently reorient into a coherent structure. In contrast, both racemic gels and non-equimolar gels exhibited significantly stronger recoverability: G′ recovered 88.79% in the racemic (5:5) system and 79.50% in the non-equimolar (*RR*-**1**:*SS*-**1** = 9:1) system after four cycles. This robust recovery can be attributed to the ability of the amorphous aggregates in racemic gels and the sea urchin-like structures in non-equimolar gels to reassemble via reversible non-covalent interactions, such as hydrogen bonding and van der Waals forces following the cessation of shear.

These rheological results conclusively demonstrate that chiral imbalance in non-equimolar mixtures modulates network topology to achieve significant enhancement of mechanical properties. The sea urchin-like architecture simultaneously improves elastic modulus, yield stress, and thixotropic recoverability, all of which are critical for supramolecular gel applications. In contrast, homochiral systems (composed of isolated crystalline fibers) and racemic systems (dominated by amorphous aggregates) represent two structural extremes that inherently limit mechanical functionality. Non-equimolar systems, by striking an optimal balance between order and connectivity, thereby enable superior overall performance.

### 2.4. Gelation Mechanism Characterization

To elucidate the molecular-level interactions governing the enantiomeric ratio-dependent gelation behavior, FT-IR spectroscopy was performed on xerogels derived from pure enantiomers, racemates, and non-equimolar mixtures (Figure S7, Table S3). In the N–H stretching region (Figure S7), all samples exhibited characteristic band shifts consistent with hydrogen-bonded urea-based gelators, aligning with previous reports [54]. The pure enantiomeric gel (*RR*-**1** or *SS*-**1**) displayed an intense, sharp peak at 3336 cm^−1^, indicative of a well-ordered hydrogen-bonding network, which correlates with the highly crystalline, isolated fibrous structures observed via SEM. As the *RR*-**1**:*SS*-**1** ratio varied from 9:1 or 1:9 toward 5:5, the N-H peak broadened and underwent a slight shift, reflecting increased diversity in hydrogen-bonding environments. The amide I band, centered around 1635 cm^−1^ (Figure S7, Table S3), remained consistent across all compositions, suggesting that the local carbonyl coordination environment remains largely unchanged regardless of chiral composition. In contrast, the amide II band (Table S3), associated with N–H bending and C–N stretching modes, shifted systematically with enantiomeric ratio: it occurred at 1589 cm^−1^ for the pure enantiomeric gel, between 1590–1591 cm^−1^ for non-equimolar gels (from 9:1/1:9 to 6:4/4:6), and at 1592 cm^−1^ for the racemic system. These shifts reflect distinct hydrogen-bonding patterns and variations in the microenvironments surrounding the amide groups.

**Figure 4:**
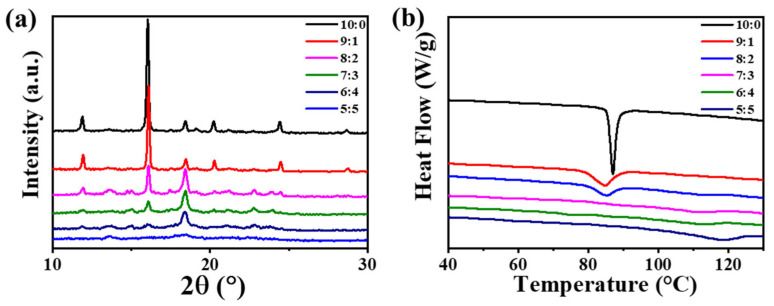
(**a**) XRD profiles of the gel samples (20 mg/mL) with different *RR*-**1**/*SS*-**1** ratios; (**b**) DSC heating curves of the gel samples (20 mg/mL) with different *RR*-**1**/*SS*-**1** ratios (heating rate: 5K min^−1^).

To investigate how chiral composition modulates the structural order of supramolecular self-assembly, X-ray diffraction (XRD) patterns were analyzed for gels with different *RR*-**1**:*SS*-**1** ratios (Figure 4a and Figure S8a). Pure enantiomeric gels (*RR*-**1** or *SS*-**1**) exhibited sharp, well-resolved diffraction peaks at 2θ≈11.9°, 16.1°, 18.5°, 20.3° and 24.5° (Figure 4a). The high intensity and narrow width of these peaks indicate a highly ordered crystalline structure with long-range periodicity, consistent with SEM observations of isolated crystalline fibers and FT-IR data that revealed well-ordered hydrogen-bonding interactions. This structural regularity originates from uniform homochiral packing, in which identical enantiomers align efficiently into a regular lattice. In contrast, as the molar ratio deviates from the pure enantiomeric composition, the XRD patterns undergo progressive changes: the intensity of the primary crystalline peak decreases and the peak broadens (Figure 4a). In the 8:2 and 2:8 systems, the peak remains prominent but becomes less sharp compared to the pure systems, indicating partial disruption of the crystalline lattice due to increasing heterochiral interactions. As the ratio approaches 5:5 (e.g., 7:3, 3:7, 6:4, 4:6), the crystalline peaks further attenuate, signaling reduced crystallinity and increased structural disorder. Residual peaks suggest the persistence of small crystalline domains, consistent with the “sea urchin-like” core-fiber architecture observed by SEM. At the equimolar ratio (5:5), well-defined crystalline peaks are entirely absent, revealing a fully amorphous structure (Figure 4a). This amorphous character aligns with SEM observations of irregular aggregates in the racemic mixtures.

Differential Scanning Calorimetry (DSC) was employed to probe the thermal transitions and melting behavior of gels formed from pure enantiomers, racemic mixtures, and non-equimolar composites (Figure 4b and Figure S8b). Pure enantiomeric gels displayed a single narrow endothermic peak at 87.0 °C (denoted as T_1_, Table S4), indicative of a homogeneous and highly crystalline structure. In contrast, racemic gels exhibited a broad endothermic peak centered at 118.0 °C (denoted as T_2_), consistent with the melting of a disordered amorphous phase. Non-equimolar gels, however, showed two distinct endothermic transitions (T_1_ and T_2_). The T_1_ values (ranging from 84.9 to 87.6 °C) closely resembled that of the pure enantiomeric gel (87.0 °C), suggesting the retention of homochiral crystalline domains similar to those in pure *RR*-**1** or *SS*-**1** systems. The T_2_ values (between 99.8 and 111.8 °C) were lower than the racemic gel transition (118.0 °C) yet higher than T_1_, indicating the coexistence of racemic-like disordered aggregates. In the 9:1/1:9 and 8:2/2:8 gels, well-defined endothermic peaks were observed at T_1_ (84.9 °C/84.3 °C and 85.2 °C/86.0 °C, respectively), accompanied by weaker signals at T_2_ (99.8 °C/100.5 °C and 112.6 °C/109.2 °C, respectively), indicating that homochiral crystalline domains constitute the dominant component. Conversely, in 7:3/3:7 and 6:4/4:6 gels, the T_2_ endotherms (111.1 °C/109.2 °C and 111.8 °C/113.8 °C, respectively) became more prominent relative to the T_1_ transitions (83.0 °C/84.9 °C and 87.6 °C/85.5 °C, respectively), suggesting that racemic-like amorphous aggregates represent the predominant structural motif in these systems.

### 2.5. Theoretical Calculation and Mechanism

To understand the morphology variations arising from different *RR*-**1**:*SS*-**1** ratios, theoretical analyses were conducted. (See computational details) Firstly, the binding energies Eb between the *RR*-**1**/*RR*-**1** and *RR*-**1**/*SS*-**1** dimers were calculated using the formula Eb = Edimer − Emonomer − Emonomer, where Edimer represents the energy of the optimized dimer, and Emonomer donates the energy of the optimized *RR*-**1**/*SS*-**1** monomer. The calculated Eb values were −150.13 kJ/mol for the *RR*-**1**/*RR*-**1** dimer and −141.66 kJ/mol for the *RR*-**1**/*SS*-**1** dimer. These results indicate that both *RR*-**1** and *SS*-**1** molecules exhibit significant binding affinity towards *RR*-**1** molecules. The optimized dimer structures revealed that both *RR*-**1**/*RR*-**1** and *RR*-**1**/*SS*-**1** dimers could be stabilized by the formation of four intermolecular hydrogen bonds.

**Figure 5:**
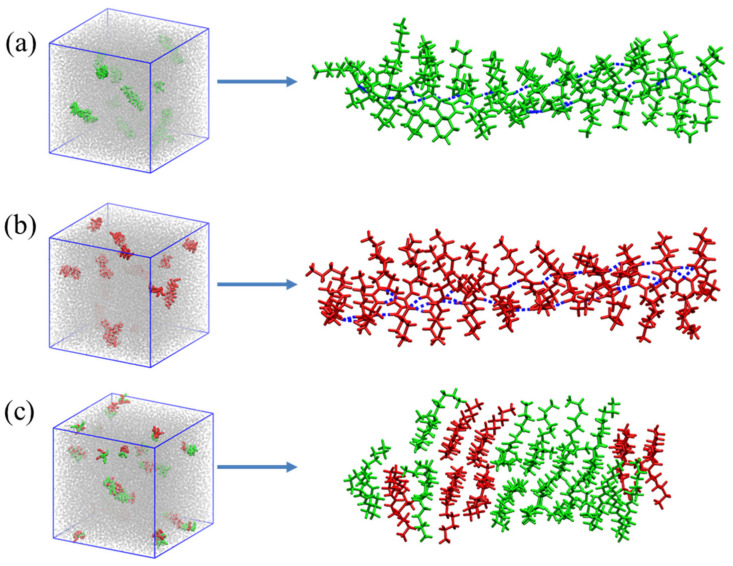
MD simulations of *RR*-**1** and *SS*-**1** in toluene (**a**) *RR*-**1**; (**b**) *SS*-**1**; (**c**) *RR*-**1**/*SS*-**1**.

To further investigate the self-assembly behaviors of *RR*-**1** and *SS*-**1** molecules in toluene, molecular dynamics (MD) simulations were performed. Systems containing homochiral *RR*-**1** and *SS*-**1** molecules, and heterochiral *RR*-**1**:*SS*-**1** (1:1) were modeled following this procedure: (1) a cubic simulation box with an initial size of 20 × 20 × 20 nm^3^ was constructed, randomly containing 100 *RR*-**1**/*SS*-**1** molecules and 19,601 toluene molecules for a solute concentration of 2% *w*/*w*; (2) energy minimization was carried out with 1 ns simulation at 300 K and 100 bar to rapidly reduce intermolecular distances; (3) systems were equilibrated at 300 K and 1 bar for 200 ns (resulting in final box sizes of approximately 15.5 × 15.5 × 15.5 nm^3^ for the three systems). In the homochiral *RR*-**1** or *SS*-**1** system (Figure 5a,b), the directional and strong hydrogen bonding interactions between identical enantiomers drive the formation of long-range ordered aggregates. In contrast, in the heterochiral system (Figure 5c), mixed aggregates comprising both *RR*-**1** and *SS*-**1** formed and stabilized by the strong intermolecular hydrogen bonds. However, due to the chiral mismatch between the two enantiomers, their binding lacks the regularity found in the homochiral system. This leads to random mixing and self-assembling of *RR*-**1** and *SS*-**1**, forming disordered aggregate nuclei with irregular morphologies.

**Figure 6:**
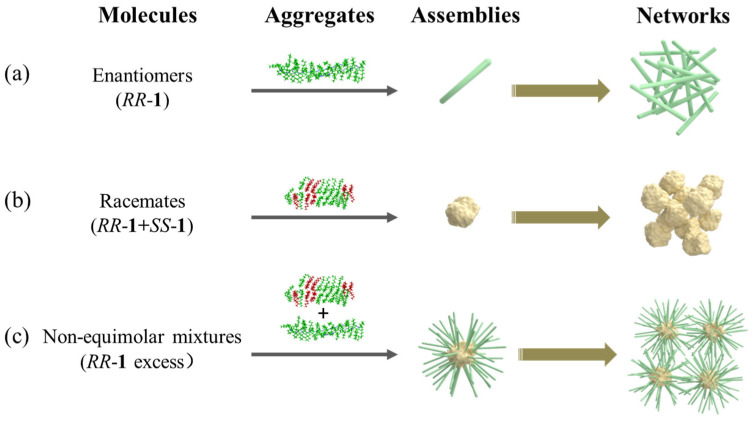
Mechanism diagram illustrating the formation of distinct hierarchical networks of gels with different *RR*-**1**/*SS*-**1** ratios: (**a**) Enantiopure *RR*-**1** gel system; (**b**) Racemic gel system; (**c**) Non-equimolar *RR*-**1**/*SS*-**1** mixed gel system.

Based on experimental results (SEM, POM, FT-IR, XRD and DSC) and theoretical calculations, we propose a mechanism underlying the distinct hierarchical networks and rheological properties of enantiomeric, racemic, and non-equimolar mixture gels (Figure 6). In homochiral systems (*RR*-**1** or *SS*-**1**), directional, strong hydrogen bonds between identical enantiomers drive the formation of single-chain aggregates. The relatively short C_6_H_13_ alkyl chains further facilitate hierarchical assembly: weak van der Waals forces and spatial packing between adjacent chains induce the initially formed single chains to gradually self-assemble into regularly packed fibrous structures, ultimately evolving into crystalline fibers with well-defined lattice order. This ordered crystallization process leads to the formation of transient fiber networks in homochiral gels, which exhibit relative weak mechanical performance (Figure 6a). In equimolar racemic systems, although *RR*-**1** and *SS*-**1** can still interact via intermolecular hydrogen bonding, chiral mismatch disrupts the regularity observed in homochiral assemblies. This results in random mixing and self-assembly of the two enantiomers into disordered aggregates with irregular morphologies, which further develop into amorphous assemblies with limited structural hierarchy, ultimately yielding a less stiff network and poor mechanical performance (Figure 6b). For non-equimolar *RR*-**1**/*SS*-**1** mixtures, the assembly process occurs in two sequential stages: first, co-assembly of *RR*-**1** and *SS*-**1** forms primary disordered aggregates that grow into critical-size nuclei. Excess *RR*-**1** or *SS*-**1** then undergoes secondary nucleation, with continuous addition of surrounding molecules driving the outgrowth of branched crystalline fibers (sea urchin-like structures) from these nuclei, eventually resulting in densely packed networks (Figure 6c). These network architectures exhibit significantly enhanced mechanical properties compared to both the transient networks of enantiomeric gels and the less stiff networks of racemic gels.

Our findings establish that a non-equimolar enantiomeric ratio represents the op-timal assembly state in our cyclohexane bisurea system, which reveals a distinct mode of chirality-directed gelation. Previous studies on chiral gelators have largely centered on comparing pure enantiomers with their racemic forms, leading to varied outcomes: Shen et al. reported that racemic bolaamphiphiles formed melamine-based hydrogels with lower CGC and higher modulus than pure enantiomers [55]. Žinić et al. showed that racemic glucosamide gelators had better gelation efficiency than single enantiomers [56]. McAulay et al. demonstrated that diastereomeric peptide gelator mixtures formed distinct nanostructures [57]. Patterson et al. observed that enantiomer blending disrupted DBS-based gels and weakened their performance [58]. Damodaran et al. found that equimolar mixtures formed novel supramolecular lattices with enhanced properties [59]. Our work demonstrates that a deliberate chiral imbalance, which is distinct from both homochiral and equimolar systems, unlocks a unique two-stage assembly pathway. This mechanism begins with heterochiral nucleation and is followed by homochiral elongation into a “sea urchin-like” network, which effectively decouples nucleation from fiber growth. It thus introduces a powerful and generalizable design principle, namely non-equimolar synergistic assembly, which substantially broadens the scope for fabricating supramolecular gels with customized hierarchical architectures and enhanced performance.

## 3. Conclusions

In summary, we have developed an enantiomeric-ratio-driven strategy to control the self-assembly and enhance the mechanical properties of cyclohexane bisurea-based supramolecular gels. Non-equimolar mixtures of *RR*-**1** and *SS*-**1** form hierarchically organized “sea urchin-like” networks with radially oriented crystalline fibers, enabling ultralow critical gelation concentrations (≈1.5 mg/mL) and significantly improved mechanical strength, exceeding homochiral gels by up to 20-fold in storage modulus and 17-fold in yield stress. This enhancement arises from a two-stage assembly mechanism initiated by heterochiral nucleation followed by crystalline fiber elongation, achieving an optimal balance between structural order and network connectivity. In contrast, homochiral systems form isolated crystalline fibers, while racemic mixtures yield amorphous aggregates with limited mechanical integrity. This work opens avenues for future studies, including exploring more non-equimolar chiral ratios to refine gel property tuning, extending the enantiomeric-ratio strategy to other low-molecular-weight gelator classes, and investigating potential applications in soft materials and functional gels.

## 4. Materials and Methods

**Materials:** (1*R*, 2*R*)-(−)-1,2-diaminocyclohexane (98%), (1*S*,2*S*)-(+)-1,2-diaminocyclohexane (98%) were purchased from Innochem (Beijing, China) and n-hexyl isocyanate (98%) were purchased from Mreda (Beijing, China). All were used as received without further purification. Tetrahydrofuran (THF, GR grade, 99.8%) and toluene (AR grade, 99.5%) were purchased from Innochem (Beijing, China) and were used as solvents as received. Potassium bromide (KBr, AR grade, 99.5%) was purchased from Innochem (Beijing, China).

**Synthesis of *RR*-1 and *SS*-1:** (1R,2R)-(−)-1,2-diaminocyclohexane (2 mmol, 1.0 eq) was dissolved in THF (500 mL) in a round-bottom flask equipped with a reflux condenser. n-Hexyl isocyanate (2.2 mmol, 2.2 eq) was added dropwise to the solution under stirring. The reaction mixture was heated to reflux (ca. 80 °C) and held for 12 h. Upon completion of the reaction (monitored by TLC), the mixture was cooled to room temperature, and the resulting gel-like precipitate was collected by filtration. The crude product was recrystallized from THF, filtered, and dried under vacuum at 30 °C for 6 h to afford *RR*-**1** as a white powder. Yield: 82.32%. ^1^H NMR (400 MHz, CDCl_3_, 25 °C): δ 5.22 (s, 2H), 4.70 (s, 2H), 3.43 (d, *J* = 8.6 Hz, 2H), 3.08 (tq, *J* = 13.2, 7.1, 6.6 Hz, 4H), 2.03 (d, *J* = 11.6 Hz, 2H), 1.72 (d, *J* = 10.5 Hz, 4H), 1.45 (q, *J* = 7.0 Hz, 4H), 1.39–1.16 (m, 16H), 0.95–0.81 (m, 6H); MS(ESI): *m*/*z*: 391.30365 [M + Na^+^]

*SS*-**1** was synthesized following an identical procedure using (1S,2S)-(+)-1,2-diaminocyclohexane as the starting material, yielding 84.48% as a white powder. ^1^H NMR (400 MHz, CDCl_3_, 25 °C): δ 5.21 (s, 2H), 4.69 (s, 2H), 3.43 (d, *J* = 8.1 Hz, 2H), 3.09 (qt, *J* = 13.2, 7.1 Hz, 4H), 2.03 (d, *J* = 11.6 Hz, 2H), 1.72 (d, *J* = 8.6 Hz, 4H), 1.45 (q, *J* = 6.9 Hz, 4H), 1.39–1.05 (m, 16H), 0.95–0.79 (m, 6H); MS(ESI): *m*/*z*: 391.30377 [M + Na^+^]

**Gelation Behavior and Critical Gel Concentration (CGC):** Gelation behavior was assessed using vial inversion tests. Specifically, 1 mL of *RR*-**1**/*SS*-**1** sample solutions (with concentrations ranging from 1 to 20 mg/mL) was sealed in glass vials. These vials were first immersed in a preheated constant-temperature thermostatic water bath set at 80 °C, held at this temperature for 10 min, and gently shaken every 2 min to ensure complete dissolution of the gelator. After the heating period, the vials were removed from the water bath, permitted to cool naturally to ambient temperature, and then left to stand undisturbed to reach a state of equilibrium. The critical gelation concentration (CGC) was defined as the minimum gelator concentration at which all three replicate vials successfully formed stable gels. A “stable gel” was confirmed by the absence of observable flow when the vials were subjected to the inversion test.

**Scanning Electron Microscopy (SEM) Observation:** Gel samples were prepared following the gelation protocol described above. A 5 μL aliquot of each gel was carefully deposited onto a pre-cleaned silicon wafer, and the samples were dried under vacuum at ambient temperature for 12 h. Prior to imaging, the dried samples were sputter-coated with a 5 nm-thick platinum layer using a Quorum Q150T ES sputter coater (Quorum Technologies Ltd., East Sussex, UK). Scanning electron microscopy (SEM) images were acquired using a Hitachi S-4800 field-emission scanning electron microscope (Hitachi, Tokyo, Japan) operated at an accelerating voltage of 10 kV.

**Polarizing Optical Microscopy (POM) Observation:** The self-assembly processes of the gelator in solutions were monitored using a Motic AE31E microscope (Motic, Xiamen, China). Gelator solution samples were placed in sealed cuvettes and heated until completely dissolved. The sealed cuvette was immediately transferred to the pre-focused stage of the microscope. The sample was allowed to cool to ambient temperature undisturbed, with real-time imaging performed at 400× magnification throughout the cooling and self-assembly process. All micrographs and videos were acquired using a Moticam S12 digital camera (Motic, Xiamen, China) connected to Motic Image Plus 3.1 software (Motic, Xiamen, China). Automatic exposure correction was deactivated to ensure consistent image quality across samples.

**Rheological Measurements:** Rheological properties were evaluated using a HAKKE MARS 40 rheometer (Thermo Fisher Scientific, Dreieich, German) equipped with a 35 mm diameter stainless steel parallel-plate geometry (gap height of 1000 μm). Prior to measurements, gel samples were carefully loaded onto the lower plate to avoid air entrapment, and excess material was carefully trimmed. Amplitude sweep tests were conducted first to determine the linear viscoelastic region (LVR), in which strain was varied from 0.1% to 100% at a fixed frequency of 1 Hz, with storage modulus (G′) and loss modulus (G″) recorded. Based on these results, a strain of 1% (within the LVR) was selected for subsequent frequency sweep tests and thixotropic assessments. For thixotropic assessments, samples were subjected to a step-strain protocol: (1) pre-shearing at 10% strain for 60 s to disrupt the network, followed by a 30 s resting period; (2) recovery at 1% strain for 60 s, followed by an additional 30 s resting period. The storage modulus recovery rate was calculated using the formula: (G′final/G′initial) × 100%. These cycles were repeated four times. All measurements were repeated three times to confirm reproducibility.

**Fourier Transform Infrared (FT-IR) Spectra:** Potassium bromide (KBr) was ground in an agate mortar and dried under an infrared lamp for 0.5 h to remove residual moisture. Xerogel samples were individually ground with the pre-dried KBr to form homogeneous mixtures, which were then compressed into transparent pellets using a hydraulic press. FT-IR spectra were acquired using a Bruker TENSOR-27 spectrometer (Bruker, Billerica, MA, USA) at a resolution of 1 cm^−1^ with 3 cumulative scans.

**Wide angle X-ray diffration:** For X-ray diffraction measurements silicon wafers with the size of 1 cm × 1 cm × 1 mm were filled with a concentrated gel (20 mg/mL), then dried in vaccum oven for 12 h. Using a D/MAX-TTRIII(CBO) X-ray diffractometer (Rigaku, Tokyo, Japan), the XRD characterization experiments of different ratios of gel samples were carried out under the conditions of wavelength 1.5481 Å, voltage 40 kV, current 200 mA, scanning speed 5°/min and scanning range 10~25°, and the XRD spectra of different ratios of gels were obtained.

**Differential Scanning Calorimetry (DSC):** About 5 mg of gel (20 mg/mL) was placed in a pre-weighed aluminium pan of Φ5.4 × 2.0 mm purchased from D&B (Shanghai, China), which was sealed and reweighed using a six-decimal-place analytical balance. All samples were measured in one-time heating scan without the cooling scan. And heating scans of 30–140 °C were performed on a TQHW-3A DSC (TA Instruments, New Castle, DE, USA) instrument at a scan rate of 5 °C/min. Following measurements, the pan was reweighed to check for potential leakage.

## Data Availability

The raw data are available from the corresponding author upon reasonable request.

## Supplementary Materials

The following supporting information can be downloaded at: https://www.mdpi.com/article/10.3390/gels11100821/s1, Figure S1: SEM images of xerogels (concentration: 10 mg/mL) obtained from *RR*-**1**/*SS*-**1** at different ratios; Figure S2: In situ POM images (concentration: 2 mg/mL) obtained from *RR*-**1**/*SS*-**1** at different ratios; Figure S3: Junction density recorded in POM images (concentration: 2 mg/mL), obtained from *RR*-**1**/*SS*-**1** at different ratios; Figure S4: Plots of rheological data; Figure S5: Plots of storage modulus G′ versus strain at different *RR*-**1**:*SS*-**1** ratios (20 mg/mL); Figure S6: Plots of storage modulus G′ versus strain at different *RR*-**1**:*SS*-**1** ratios (5 mg/mL); Figure S7: Infrared (IR) spectra of the gel samples at different *RR*-**1**:*SS*-**1** ratios (concentration: 20 mg/mL); Figure S8: (a) XRD profiles of the gel samples (20 mg/mL) at different *RR*-**1**/*SS*-**1** ratios; (b) DSC heating curves of the gel samples (20 mg/mL) at different *RR*-**1**/*SS*-**1** ratios (heating rate: 5 K min^−1^); Figure S9: DSC heating and cooling curves of (a) tolune; (b,c) leaking samples (heating rate: 5 K min^−1^); Table S1: Critical gelation concentration of *RR***-1**/*SS*-**1** at different ratios; Table S2: Junction density of the gel samples at different *RR*-**1**:*SS*-**1** ratios in POM; Table S3: Selected FT-IR Characteristic Bands (Wavenumber, ν; Unit: cm^−1^) for gel samples at different *RR*-**1**:*SS*-**1** ratios; Table S4: The thermal transitions behavior of gel samples at different *RR*-**1**/*SS*-**1** ratios; Video S1: POM of 10:0; Video S2: POM of 9:1; Video S3: POM of 5:5. References [60,61,62,63,64,65] are cited in the Supplementary Materials.

## Abbreviations

The following abbreviations are used in this manuscript:

*RR*-**1**	1,1’-((1R,2R)-cyclohexane-1,2-diyl)bis(3-hexylurea)
*SS*-**1**	1,1’-((1S,2S)-cyclohexane-1,2-diyl)bis(3-hexylurea)
