# Supplementary material for: Enantiomeric Ratio Modulates Hierarchical Networks and Rheological Performance in Cyclohexane Bisurea Supramolecular Gels
Source: Gels. 2025 Oct 13;11(10):821. doi: 10.3390/gels11100821 (PMC12563907; doi:10.3390/gels11100821)
Supplement: Supplementary file 1 [file gels-11-00821-s001.zip › gels-3887923-supplementary.pdf]

Supplementary Materials

Enantiomeric Ratio Modulates Hierarchical Networks and Rheological Performance in Cyclohexane Bisurea Supramolecular Gels

Shaoshuai Hua, Yuqian Jiang *, Andong Song and Jian Jiang *

CAS Key Laboratory of Nanosystem and Hierarchical Fabrication, National Center for Nanoscience and Technology, Beijing 100190, China

* Correspondence: jiangyq@nanoctr.cn (Y.J.); jiangj@nanoctr.cn (J.J.)

Computational Details

The geometries of both monomers and dimers geometries were optimized via density functional theory (DFT) at the B3LYP-D3(BJ)/6-311+G(d,p) level using the Gaussian16 package [60]. Molecular dynamics (MD) simulations were performed using GROMACS-2019.3 software package with a modified general AMBER force field [61–62]. The force field parameters for equilibrium bond lengths and angles were updated based on the optimized geometries, which were obtained by density functional theory (DFT) at the B3LYP-D3(BJ)/6-311+G(d,p) level. The atomic charges were calculated by DFT at the B3LYP/6-311+G(d,p) level combined with the restrained electrostatic potential fitting scheme [63]. A spherical cut-off of 1.2 nm for the summation of van der Waals interactions and short-range Coulomb interactions and the particle-mesh Ewald method for solving long-range Coulomb interactions were used throughout. The simulations were carried out under 3D periodic boundary conditions using the leap-frog integrator with a time step of 1.0 fs. The velocity rescaling thermostat and the Berendsen barostat under the NPT ensemble were applied to control the temperature and pressure, respectively [64,65].

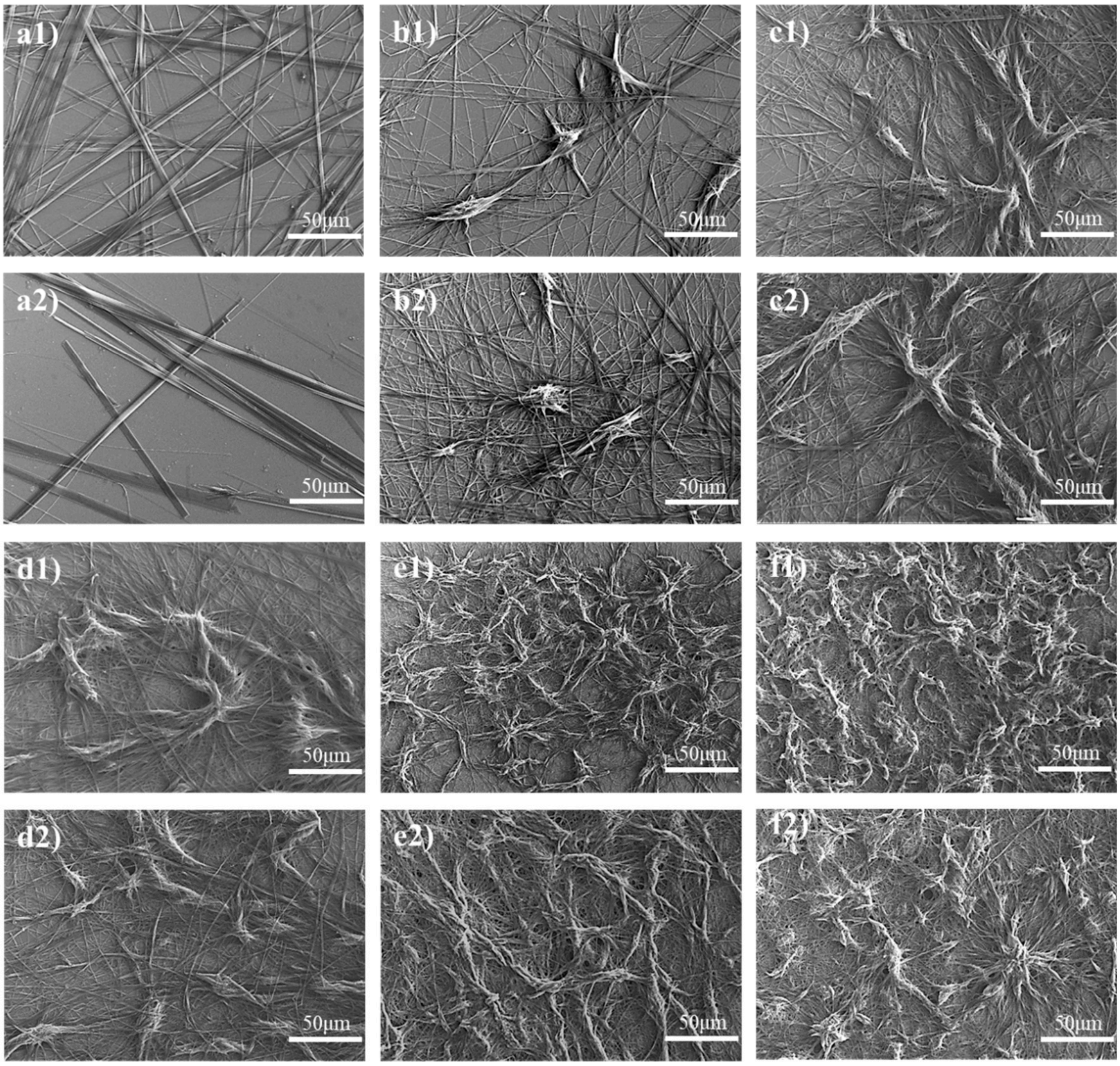

Figure S1. SEM images of xerogels (concentration: 10 mg/mL) obtained from *RR*-**1**/*SS*-**1** at different ratios: a1)-e1) represent the ratios of 10:0, 9:1, 8:2, 7:3, 6:4, while a2)-e2) represent their corresponding enantiomeric counterpart ratios; f) represents the *RR*-**1**: *SS*-**1** = 5:5 (racemic mixture).

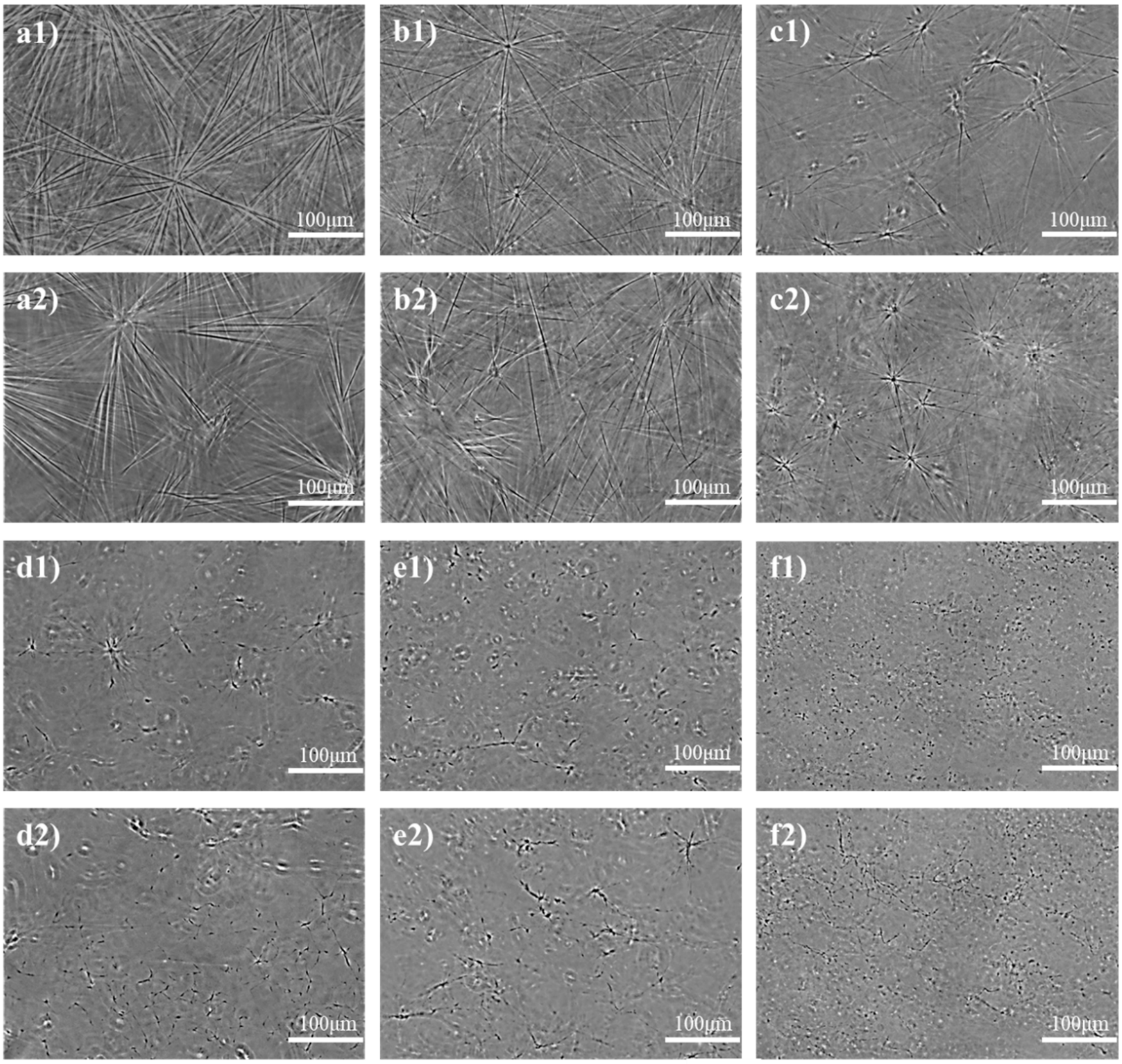

Figure S2. In situ POM images (concentration: 2 mg/mL), obtained from *RR*-**1**/*SS*-**1** at different ratios: a1)-e1) represent the ratios of 10:0, 9:1, 8:2, 7:3, 6:4, while a2)-e2) represent their corresponding enantiomeric counterpart ratios; f) represents the *RR*-**1**: *SS*-**1** = 5:5 (racemic mixture).

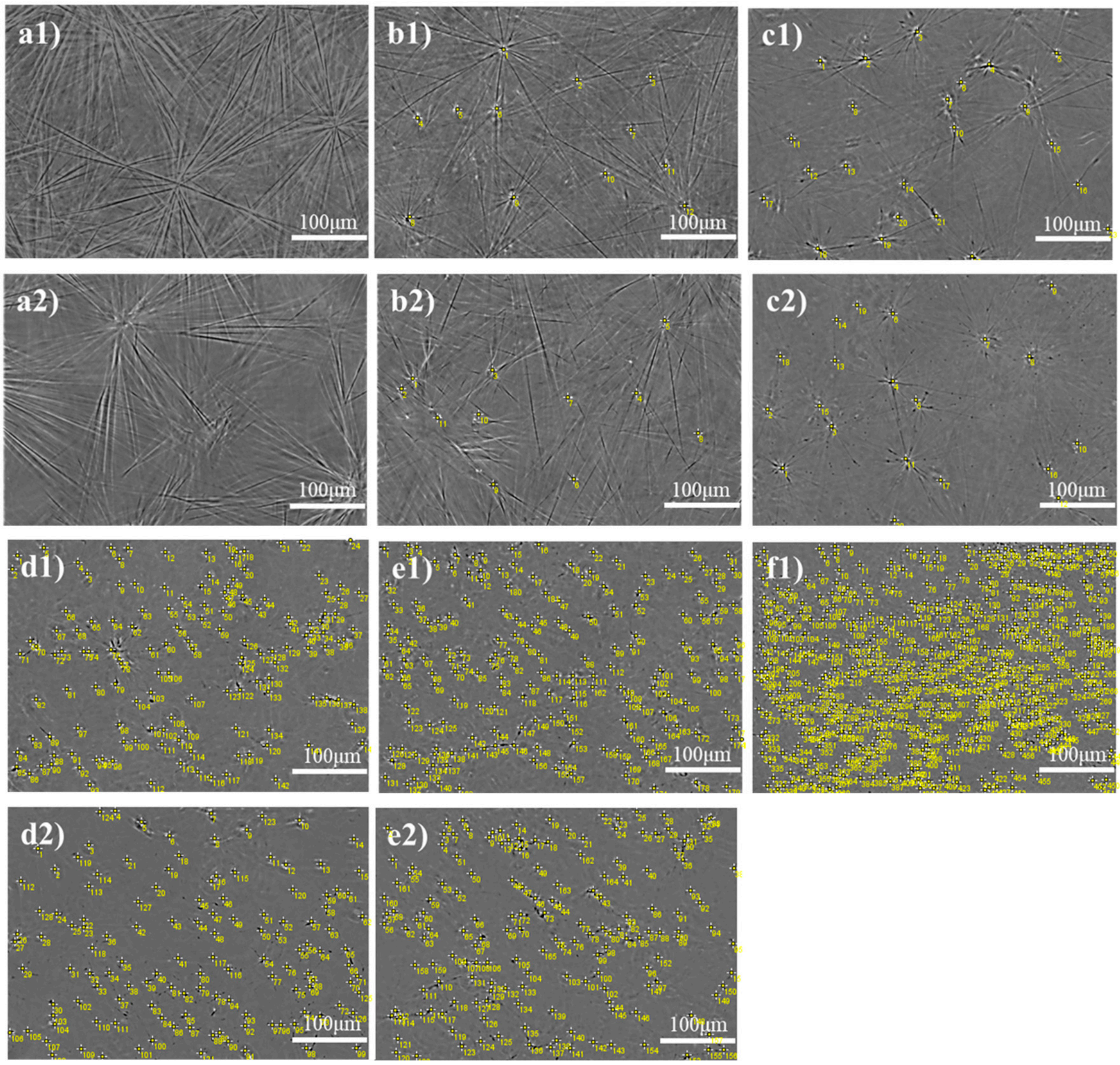

Figure S3. Junction density recorded in POM images (concentration: 2 mg/mL), obtained from *RR*-1/*SS*-1 at different ratios: a1)-e1) represent the ratios of 10:0, 9:1, 8:2, 7:3, 6:4; a2)-e2) represent their corresponding enantiomeric counterpart ratios, f) represents the *RR*-1: *SS*-1 = 5:5 (racemic mixture)

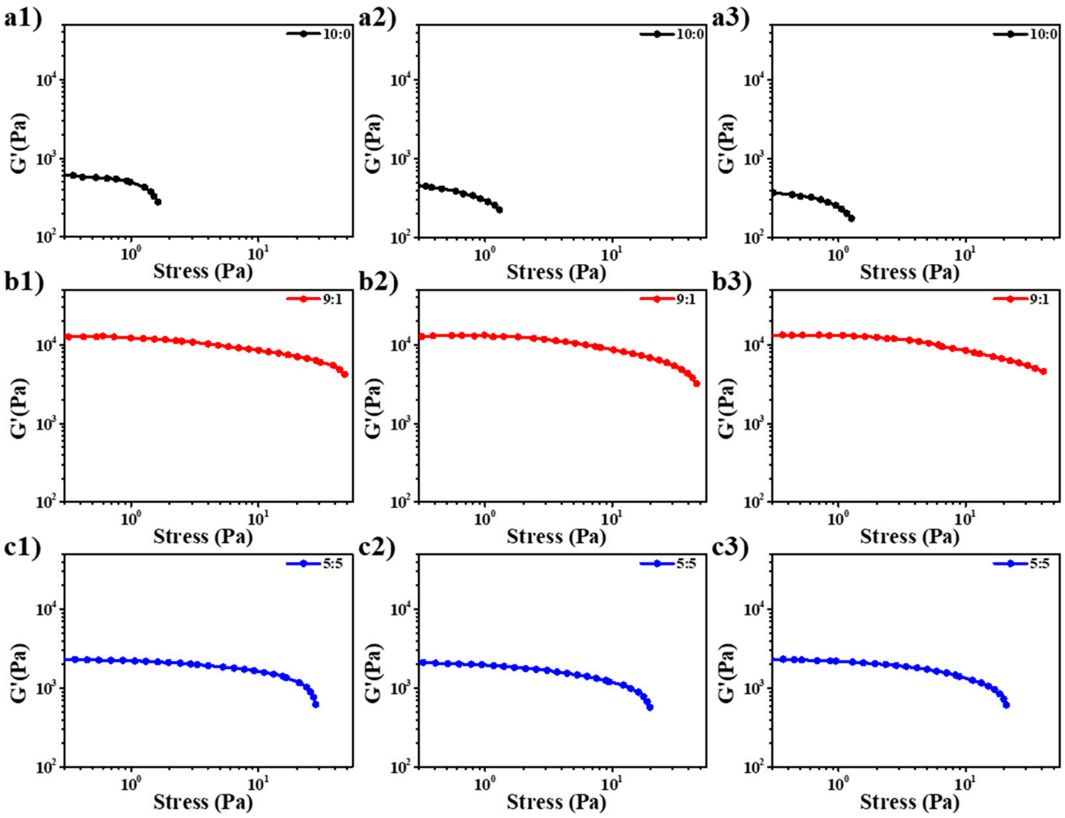

Figure S4.1 Plots of storage modulus G′ versus stress at different *RR*-1: *SS*-1 ratios(20mg/mL): a1)-a3) represent the ratios of 10:0; b1)-b3) represent the ratios of 9:1; c1)-c3) represent the ratios of 5:5.

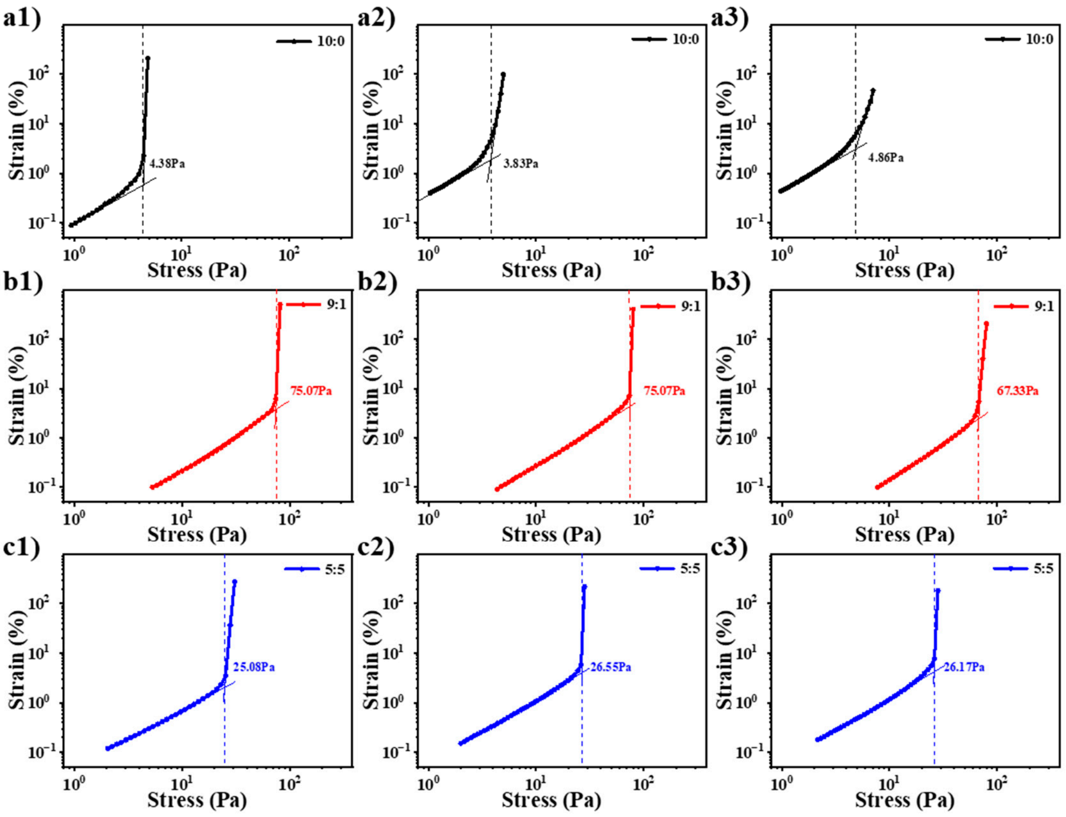

Figure S4.2 Plots of yield stress under strain at different *RR*-1:*SS*-1 ratios(20mg/mL): a1)-a3) represent the ratios of 10:0; b1)-b3) represent the ratios of 9:1; c1)-c3) represent the ratios of 5:5.

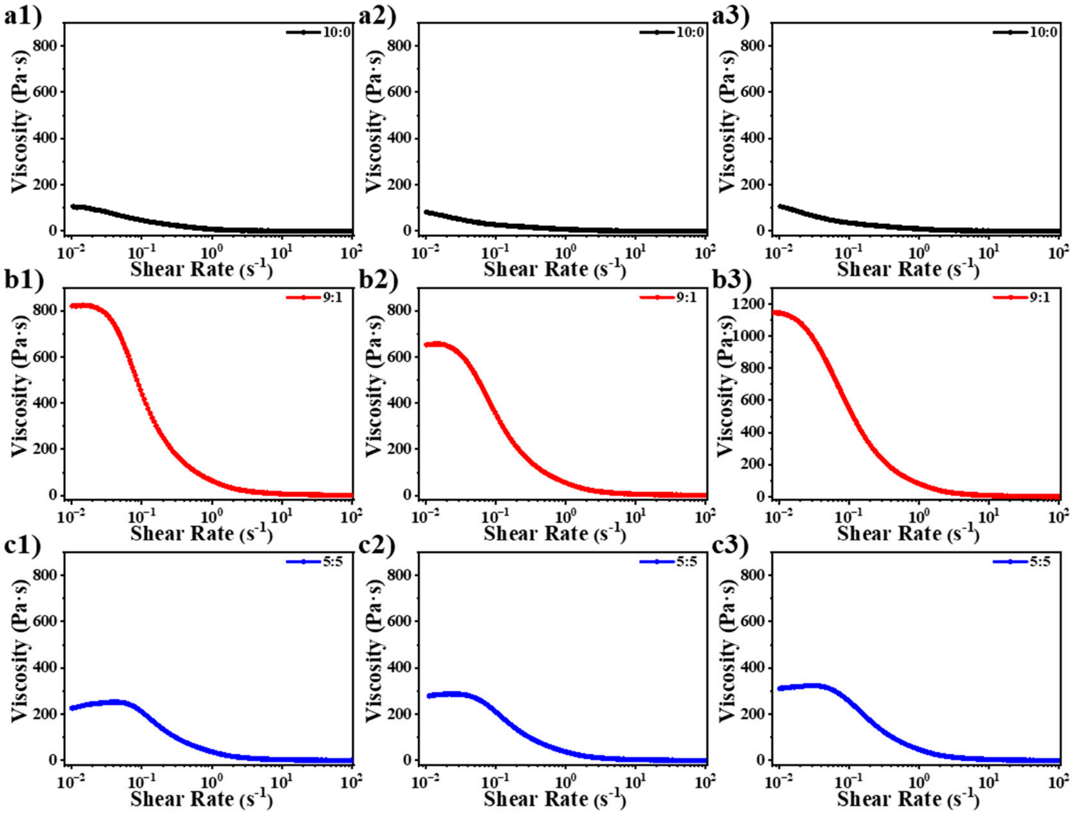

Figure S4.3 Plots of viscosity versus shear rate at different *RR*-1:*SS*-1 ratios(20mg/mL): a1)-a3) represent the ratios of 10:0; b1)-b3) represent the ratios of 9:1; c1)-c3) represent the ratios of 5:5.

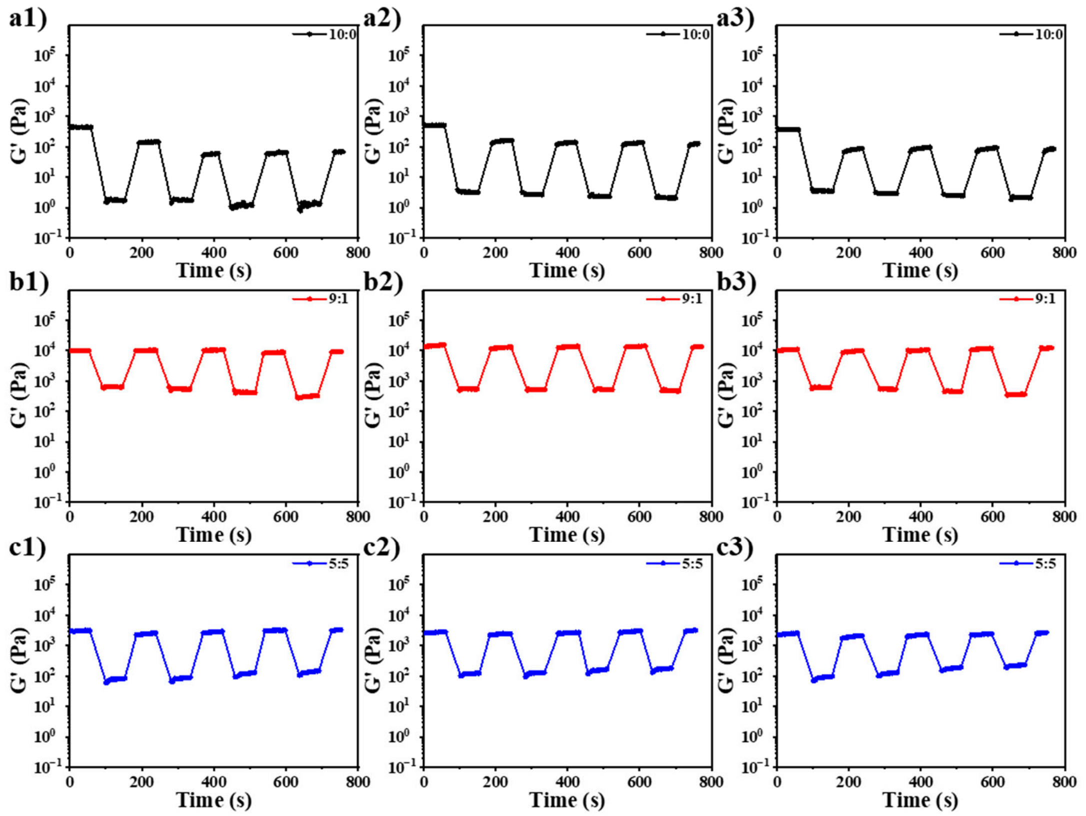

Figure S4.4 shear recovery tests for three gel systems at different *RR*-1:*SS*-1 ratios(20mg/mL): a1)-a3) represent the ratios of 10:0; b1)-b3) represent the ratios of 9:1; c1)-c3) represent the ratios of 5:5.

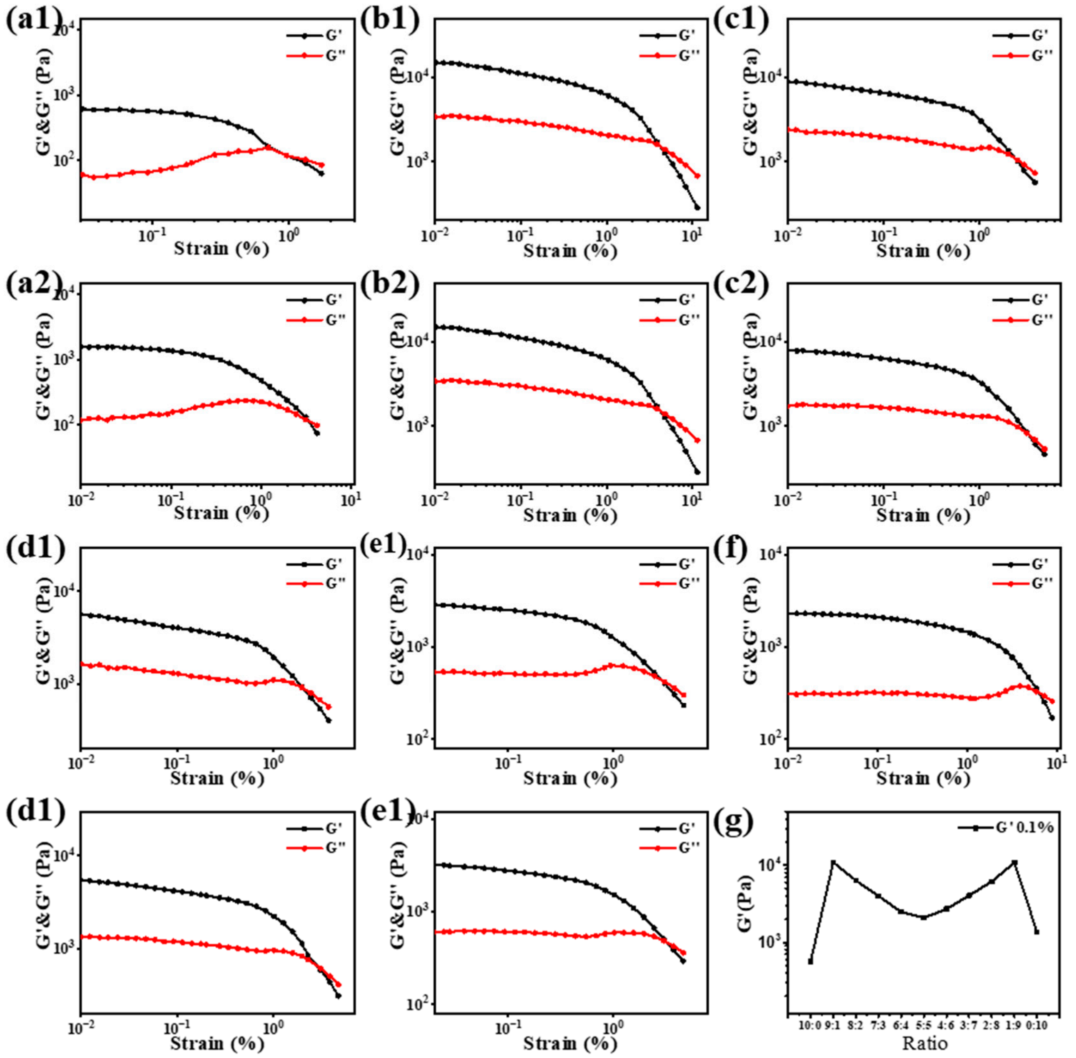

Figure S5. Plots of storage modulus G′ versus strain at different *RR*-1:*SS*-1 ratios(20mg/mL): a1)-e1) represent the ratios of 10:0, 9:1, 8:2, 7:3, 6:4; a2)-e2) represent their corresponding enantiomeric counterpart ratios; f) represents the *RR*-**1**:*SS*-**1** = 5:5 (racemic mixture); g)represent the G′ at 0.1% of all ratios.

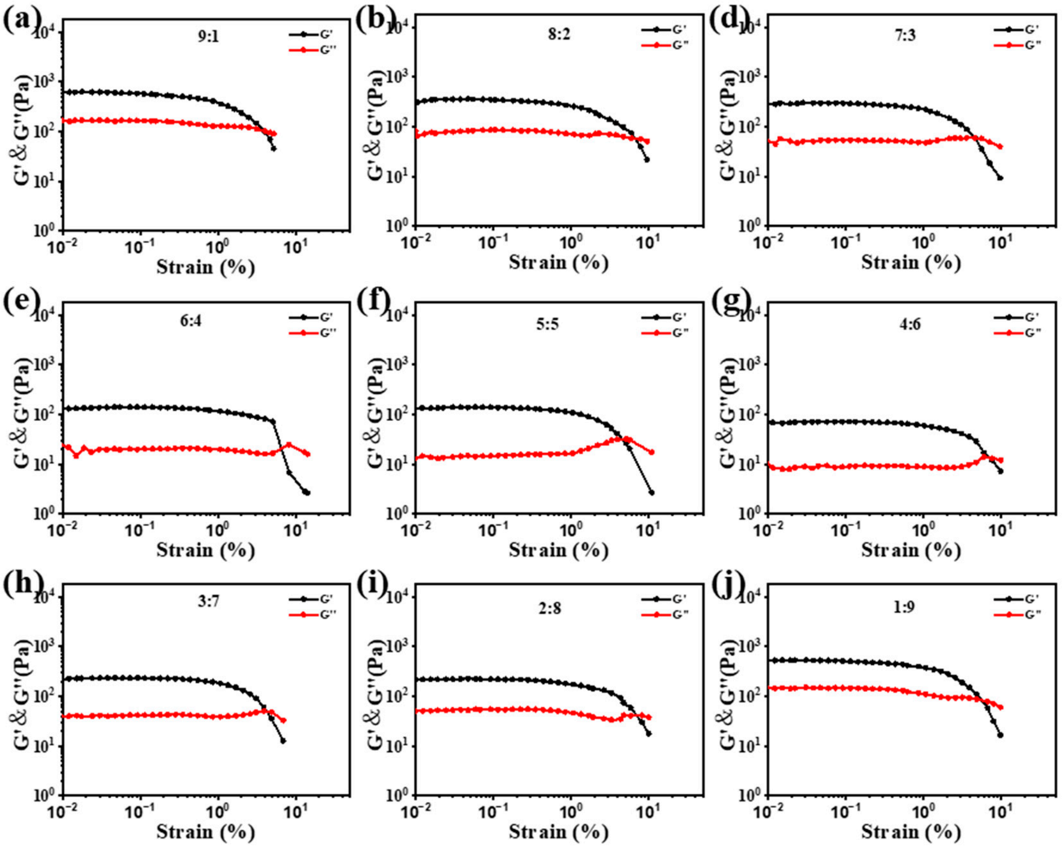

Figure S6. Plots of storage modulus G′ versus strain at different *RR*-1: *SS*-1 ratios(5mg/mL): a)-i) represent the ratios of 9:1, 8:2, 7:3, 6:4, 5:5, 4:6, 3:7, 2:8, 1:9.

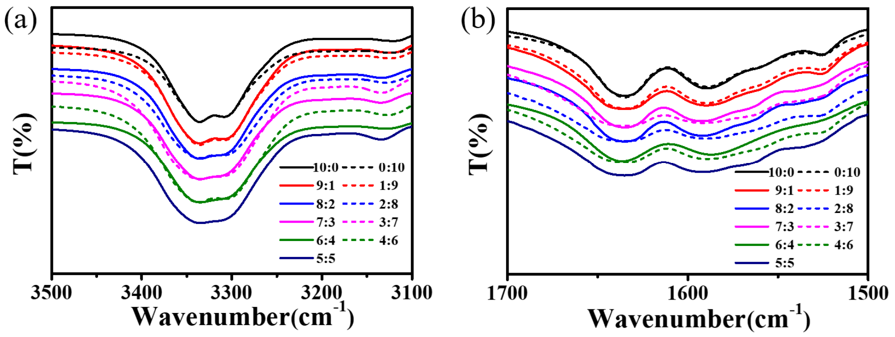

Figure S7. Infrared (IR) spectra of the gel samples at different *RR*-**1**: *SS*-**1** ratios (concentration: 20 mg/mL).

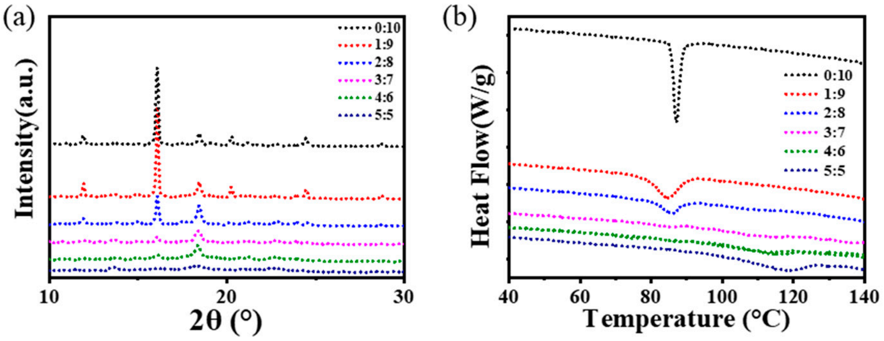

Figure S8 (a) XRD profiles of the gel samples (concentration: 20 mg/mL) with different *RR*-**1**/*SS*-**1** ratios; (b) DSC heating curves of the gel samples (concentration: 20 mg/mL) with different RR-1/SS-1 ratios (heating rate: 2 K min^{-1}).

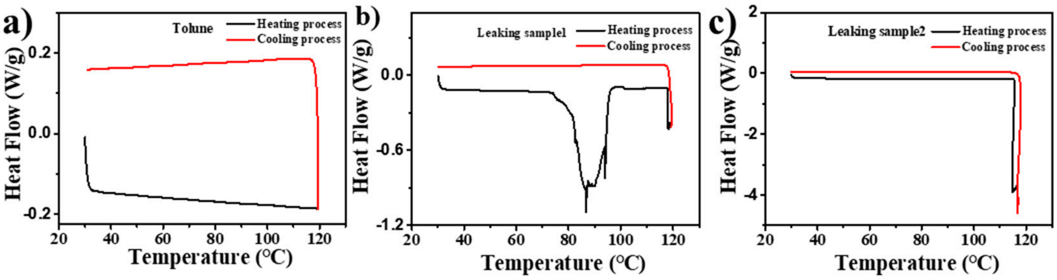

Figure S9 DSC heating and cooling curves of a) tolune; b) c) leaking samples (heating rate: 5 K min^{-1})

Table S1. Critical gelation concentration of *RR*-**1**/*SS*-**1** at different ratios

Entry	Ratio (*RR*-**1**:*SS*-**1**)	Concentration (mg/mL)
1	10:0(0:10)	10.0
2	9:1(1:9)	1.5
3	8:2(2:8)	1.5
4	7:3(3:7)	2.5
5	6:4(4:6)	3.0
6	5:5	4.0

Table S2. Junction density of the gel samples at different *RR*-1:*SS*-1 ratios in POM.

Entry	Ratio (*RR*-1:*SS*-1)	Number of Junctions	Junction density/mm^{-2}
1	10:0	0	0
2	9:1	12	73
3	8:2	23	140
4	7:3	142	866
5	6:4	181	1104
6	5:5	457	2787
7	4:6	165	1006
8	3:7	128	781
9	2:8	20	122
10	1:9	11	67
11	0:10	0	0

Table S3. Selected FT-IR Characteristic Bands (Wavenumber, ν; Unit: cm^{-1}) for gel samples with different *RR*-**1**: *SS*-**1** ratios.

Entry	Ratio (*RR*-1:*SS*-1)	N-H stretch	AmideI	AmideII
1	10:0(0:10)	3336	1635	1599
2	9:1(1:9)	3336	1635	1590
3	8:2(2:8)	3336	1635	1591
4	7:3(3:7)	3336	1635	1592
5	6:4(4:6)	3337	1635	1589
6	5:5	3335	1635	1592

Table S4. The thermal transitions behavior of gel samples with different *RR*-**1**/*SS*-**1** ratios.

Entry	Ratio (*RR*-1:*SS*-1)	T_1(°C)	T_2(°C)
1	10:0	87.0	/
2	9:1	84.9	99.8
3	8:2	85.2	112.6
4	7:3	83.0	111.1
5	6:4	87.6	111.8
6	5:5	/	111.8
7	4:6	85.5	113.8
8	3:7	84.9	109.2
9	2:8	86.0	109.2
10	1:9	84.3	100.5
11	0:10	87.1	/

60. Gaussian 16, Revision C.01, Frisch, M. J.; Trucks, G. W.; Schlegel, H. B.; Scuseria, G. E.; Robb, M. A.; Cheeseman, J. R.; Scalmani, G.; Barone, V.; Petersson, G. A.; Nakatsuji, H.; Li, X.; Caricato, M.; Marenich, A. V.; Bloino, J.; Janesko, B. G.; Gomperts, R.; Mennucci, B.; Hratchian, H. P.; Ortiz, J. V.; Izmaylov, A. F.; Sonnenberg, J. L.; Williams-Young, D.; Ding, F.; Lipparini, F.; Egidi, F.; Goings, J.; Peng, B.; Petrone, A.; Henderson, T.; Ranasinghe, D.; Zakrzewski, V. G.; Gao, J.; Rega, N.; Zheng, G.; Liang, W.; Hada, M.; Ehara, M.; Toyota, K.; Fukuda, R.; Hasegawa, J.; Ishida, M.; Nakajima, T.; Honda, Y.; Kitao, O.; Nakai, H.; Vreven, T.; Throssell, K.; Montgomery, J. A., Jr.; Peralta, J. E.; Ogliaro, F.; Bearpark, M. J.; Heyd, J. J.; Brothers, E. N.; Kudin, K. N.; Staroverov, V. N.; Keith, T. A.; Kobayashi, R.; Normand, J.; Raghavachari, K.; Rendell, A. P.; Burant, J. C.; Iyengar, S. S.; Tomasi, J.; Cossi, M.; Millam, J. M.; Klene, M.; Adamo, C.; Cammi, R.; Ochterski, J. W.; Martin, R. L.; Morokuma, K.; Farkas, O.; Foresman, J. B.; Fox, D. J. Gaussian, Inc., Wallingford CT, **2016**.

61. Páll, S.; Zhmurov, A.; Bauer, P.; Abraham, M.; Lundborg, M.; Gray, A.; Hess, B.; Lindahl, E., Heterogeneous parallelization and acceleration of molecular dynamics simulations in GROMACS. *J. Chem. Phys.* **2020**, *153*, 134110. https://doi.org/10.1063/5.0018516.

62. Wang, J.; Wolf, R. M.; Caldwell, J. W.; Kollman, P. A.; Case, D. A., Development and testing of a general amber force field. *J. Comput. Chem.* **2004**, *25*, 1157-1174. https://doi.org/10.1002/jcc.20035.

63. Bayly, C. I.; Cieplak, P.; Cornell, W.; Kollman, P. A., A Well-Behaved Electrostatic Potential based Method Using Charge Restraints for Deriving Atomic Charges: The RESP Model. *J. Phys. Chem.* **1993**, *97*, 10269-10280. https://doi.org/10.1021/j100142a004.

64. Bussi, G.; Donadio, D.; Parrinello, M., Canonical sampling through velocity rescaling. *J. Chem. Phys.* **2007**, *126*, 014101. https://doi.org/10.1063/1.2408420.

65. Berendsen, H. J. C.; Postma, J. P. M.; van Gunsteren, W. F.; DiNola, A.; Haak, J. R., Molecular Dynamics with Coupling to an Axternal Bath. *J. Chem. Phys.* **1984**, *81*, 3684-3690.
